# Preovulatory serum estradiol concentration is positively associated with oocyte ATP and follicular fluid metabolite abundance in lactating beef cattle

**DOI:** 10.1093/jas/skac136

**Published:** 2022-07-01

**Authors:** Casey C Read, J Lannett Edwards, F Neal Schrick, Justin D Rhinehart, Rebecca R Payton, Shawn R Campagna, Hector F Castro, Jessica L Klabnik, Sarah E Moorey

**Affiliations:** Department of Animal Science, University of Tennessee, Knoxville, TN 37996, USA; Department of Animal Science, University of Tennessee, Knoxville, TN 37996, USA; Department of Animal Science, University of Tennessee, Knoxville, TN 37996, USA; Department of Animal Science, University of Tennessee, Knoxville, TN 37996, USA; Department of Animal Science, University of Tennessee, Knoxville, TN 37996, USA; Department of Chemistry, University of Tennessee, Knoxville, TN 37996, USA; Department of Chemistry, University of Tennessee, Knoxville, TN 37996, USA; Department of Animal Science, University of Tennessee, Knoxville, TN 37996, USA; Department of Animal Science, University of Tennessee, Knoxville, TN 37996, USA

**Keywords:** cumulus–oocyte complex metabolism, estradiol, follicular fluid metabolome, induced ovulation, preovulatory follicle, progesterone

## Abstract

Cattle induced to ovulate a small, physiologically immature preovulatory follicle had reduced oocyte developmental competence that resulted in decreased embryo cleavage and day 7 embryo quality compared with animals induced to ovulate a more advanced follicle. RNA-sequencing was performed on oocytes and their corresponding cumulus cells approximately 23 h after gonadotropin-releasing hormone (**GnRH**) administration to induce the preovulatory gonadotropin surge suggested reduced capacity for glucose metabolism and oxidative phosphorylation in the cumulus cells and oocytes from follicles ≤11.7 mm, respectively. We hypothesized that induced ovulation of a small, physiologically immature preovulatory follicle results in a suboptimal follicular microenvironment and reduced oocyte metabolic capacity. We performed a study with the objective to determine the impact of preovulatory follicle diameter and serum estradiol concentration at GnRH administration on oocyte metabolic competence and follicular fluid metabolome profiles. We synchronized the development of a preovulatory follicle and collected the follicle contents via transvaginal aspiration approximately 19 h after GnRH administration in lactating beef cows (*n* = 319). We determined ATP levels and mitochondrial DNA (**mtDNA**) copy number in 110 oocytes and performed ultra-high-performance liquid chromatography–high resolution mass spectrometry metabolomic studies on 45 follicular fluid samples. Intraoocyte ATP and the amount of ATP produced per mtDNA copy number were associated with serum estradiol concentration at GnRH and time from GnRH administration to follicle aspiration (*P* < 0.05). mtDNA copy number was not related to follicle diameter at GnRH, serum estradiol concentration at GnRH, or any potential covariates (*P* > 0.10). We detected 90 metabolites in the aspirated follicular fluid. We identified 22 metabolites associated with serum estradiol concentration at GnRH and 63 metabolites associated with follicular fluid progesterone concentration at the time of follicle aspiration (FDR < 0.10). Pathway enrichment analysis of significant metabolites suggested altered proteinogenesis, citric acid cycle, and pyrimidine metabolism in follicles of reduced estrogenic capacity pre-gonadotropin surge or reduced progesterone production by the time of follicle aspiration.

## Teaser

Results from this study highlight the importance of the preovulatory physiological maturity of the follicle at the time of the preovulatory gonadotropin surge on oocyte metabolic capacity and follicular fluid metabolome profiles following approximately 17 h of in vivo maturation.

## Introduction

Utilization of fixed-time artificial insemination positively impacts reproductive management and genetic merit of the beef herd (Lima et al., 2010; Sá Filho et al., 2010; Funston et al., 2012; Rodgers et al., 2012; Cushman et al., 2013; Lamb and Mercadante, 2016; Baruselli et al., 2018). Such protocols, however, result in a subset of cattle that have not displayed estrus prior to insemination and rely on the administration of gonadotropin-releasing hormone (**GnRH**) to induce ovulation. Expression of estrus is essential for optimal pregnancy outcomes, and induced ovulation of small, physiologically less advanced preovulatory follicles is associated with decreased pregnancy rates and increased early pregnancy loss in beef cattle (Lamb et al., 2001; Perry et al., 2005, 2007). Reduced fertility is likely the result of ovulation of an oocyte with reduced developmental competence and poor preparation of the maternal environment to support a pregnancy (Perry et al., 2005; Sá Filho et al., 2010; Atkins et al., 2013; Jinks et al., 2013; Ciernia et al., 2021). Cattle induced to ovulate prematurely after pharmacological induction of ovulation had lower serum estradiol concentrations before GnRH administration and reduced progesterone production by the corpus luteum (**CL**) after ovulation compared with animals that displayed estrus (Busch et al., 2008; Richardson et al., 2016; Cooke et al., 2019). Additionally, cattle that were pharmacologically induced to ovulate a small (<12.5 mm) vs. large (≥12.5 mm) preovulatory follicle had impaired oocyte developmental competence that resulted in a decreased probability of producing a cleaved embryo and decreased embryo quality grade 7 d after insemination (Atkins et al., 2013). In the same study, serum estradiol concentration at the time of GnRH administration to induce ovulation was also positively associated with the probability of recovering a cleaved or high-quality embryo. Multiple in vitro studies have demonstrated that removal of the oocyte from the follicle, and thus inducing oocyte maturation, prior to the endogenous gonadotropin surge results in decreased oocyte developmental competence as evidenced by decreased cleavage rates, blastocyst rates, and embryo quality (Pavlok et al., 1992; Lonergan et al., 1994; Blondin and Sirard, 1995; Fair et al., 1995; Blondin et al., 1996; Hyttel et al., 1997; Otoi et al., 1997; Hagemann et al., 1999; van de Leemput et al., 1999; Dieleman et al., 2002; Iwata et al., 2004). Collectively, these studies support the hypothesis that decreased oocyte developmental competence contributes to the reduced fertility in cattle induced to ovulate a small, physiologically immature preovulatory follicle.

Though oocytes obtain meiotic competence much earlier than the preovulatory follicle stage of development, follicles greater than approximately 9 mm contain oocytes that are completing the capacitation phase of oogenesis (Hyttel et al., 1997; Mermillod et al., 1999; Conti and Franciosi, 2018). Throughout this phase, the oocyte is maintained in a state of meiotic arrest while it undergoes ultrastructural changes, modifies its RNA and proteins to prevent degradation, increases its organelle number, alters the distribution of organelles, and finishes accumulating nutrient and metabolic substrate stockpiles important to sustain development (Hyttel et al., 1986, 1989, 1997; Mermillod et al., 1999; Merton et al., 2003; Swain and Pool, 2008; Gougeon, 2010; Zhang and Smith, 2015; Labrecque et al., 2016; Liu et al., 2017; Reader et al., 2017; Conti and Franciosi, 2018). Metabolism in the oocyte and early embryo relies on energetic substrates such as pyruvate, lactate, NADH, and FADH_2_ that are derived from cumulus cell glycolytic activity and transferred to the oocyte via gap junctions during antral follicle development (Sutton-McDowall et al., 2010; Winterhager and Kidder, 2015; Richani et al., 2021). The oocyte creates stockpiles of these substrates that are utilized by its mitochondria to produce ATP necessary to sustain development through the energy-demanding processes of oocyte maturation, fertilization, and embryo development to the blastocyst stage (Johnson et al., 2007; Swain and Pool, 2008; Chappel, 2013; Zhang and Smith, 2015). In addition to metabolic coupling with the cumulus cells, the follicular microenvironment plays a key role in the ability of the oocyte to achieve metabolic competency. The follicular fluid of antral follicles provides many of the nutrients, metabolic compounds, and signaling molecules that are essential for the cumulus cells to perform glycolysis and for the oocyte to increase its mitochondrial number and perform oxidative phosphorylation (Jeong et al., 2009; Hennet and Combelles, 2012; Mao et al., 2012; Dunning et al., 2014; Da Broi et al., 2018; Aguila et al., 2020).

The preovulatory gonadotropin surge induces oocyte maturation, and the subsequent breakdown of gap junctional transfer of metabolic compounds to the oocyte (Granot and Dekel, 2002; Conti et al., 2012; Conti and Franciosi, 2018). By inducing the preovulatory gonadotropin surge before estrus and the onset of an endogenous gonadotropin surge, the capacitation timeline of the oocyte is interrupted. As such, we have previously detected transcriptome profiles indicative of altered oxidative phosphorylation or mitochondrial function in oocytes and altered glycolytic activity in cumulus cells collected from small preovulatory follicles approximately 23 h following GnRH administration to induce the preovulatory gonadotropin surge (Moorey et al., 2021). We recently also detected a positive correlation between preovulatory follicle diameter and abundance of metabolites involved in glucose metabolism in follicular fluid collected approximately 20 h following an induced gonadotropin surge of preovulatory follicles of increasing size (Read et al., 2021b). Such observations may be due to incomplete acquisition of the follicle’s physiological maturity before the induced preovulatory gonadotropin surge and thus an altered follicular microenvironment and inadequate opportunity for the oocyte to complete capacitation. Therefore, we hypothesized that the follicle’s developmental progression toward estrus and exposure to an endogenous preovulatory gonadotropin surge leads to necessary alterations in the follicular microenvironment that are essential for optimal metabolic capacity of the cumulus–oocyte complex (**COC**), and induced ovulation of small, physiologically immature preovulatory follicles resulting in a suboptimal follicular microenvironment and reduced oocyte metabolic capacity. To test this hypothesis, we utilized preovulatory follicle diameter and serum estradiol concentration as indicators of follicle physiological maturity at the time of GnRH administration to induce the preovulatory gonadotropin surge. We then performed a study with the objective to determine relationships between preovulatory follicle physiological maturity at the time of GnRH administration and subsequent follicular fluid metabolome profiles, oocyte mitochondrial DNA (**mtDNA**) copy number, and oocyte ATP concentration.

## Materials and Methods

### Animal handling and synchronization of preovulatory follicle development

**Figure 1.**
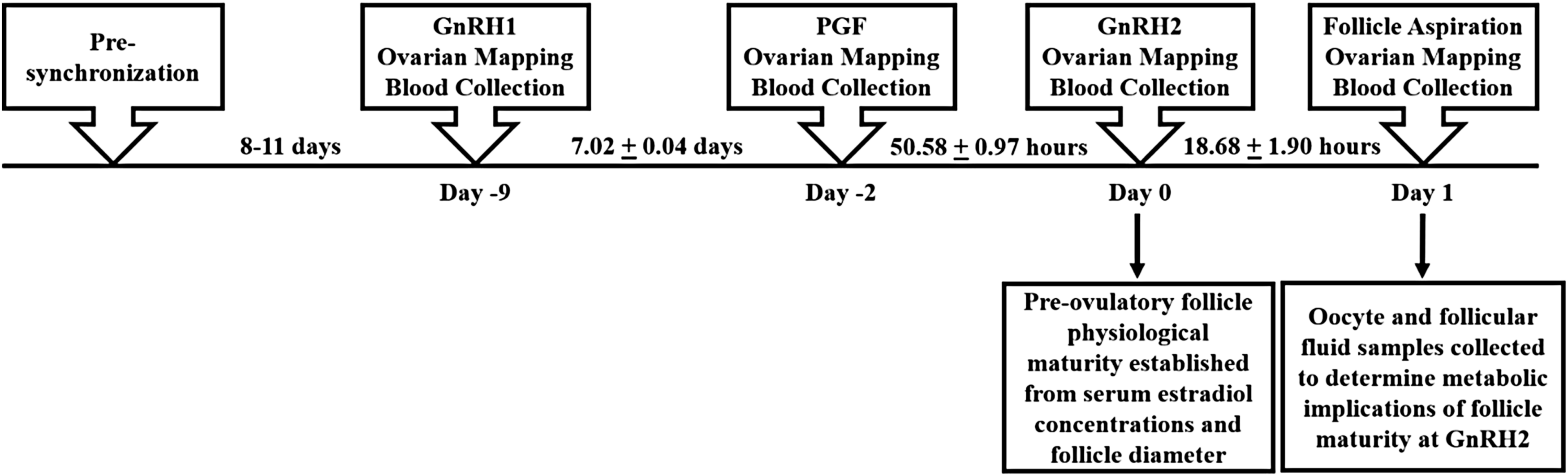
Timeline for synchronization of preovulatory follicle development and sample collection. We collected oocyte and follicular fluid samples approximately 19 h after GnRH administration to induce the preovulatory gonadotropin surge to determine the functional significance of varied preovulatory follicle maturity at the time of GnRH administration on the follicular fluid metabolome and oocyte metabolic capacity. GnRH1, gonadotropin-releasing hormone administration to turn over a new follicular wave at the onset of synchronization; PGF, prostaglandin F_2α_; GnRH2, gonadotropin-releasing hormone administration to induce the preovulatory gonadotropin surge. Values are depicted as mean ± SD.

All animal protocols and procedures were approved by the University of Tennessee Knoxville Institutional Animal Care and Use Committee. Preovulatory follicle development was synchronized in postpartum, suckled beef cattle (Angus; *n* = 319) according to procedures outlined in Figure 1. The study contained two replicates conducted over 2 yr (year 1: *n* = 162, year 2: *n* = 157). Estrous cycles were presynchronized by administration of GnRH (Cystorelin, Boehriner Ingelheim, Ingelheim am Rhein, Germany) and placement of a controlled internal drug release (**CIDR**; Eazi-Breed CIDR, Zoetis Animal Health, Kalamazoo, MI, USA). After 7 d, the CIDR was removed and cows were administered prostaglandin F_2α_ (**PGF**; Lutalyse HighCon, Zoetis Animal Health). Approximately 66 h later, cows were administered a second dosage of GnRH. Cows were then divided into 4 groups to facilitate transvaginal aspiration with 21 to 44 animals per group. Eight to eleven days after presynchronization, cows were administered GnRH on day −9 (**GnRH1**) to start a new follicular wave. On day −2, PGF was administered to lyse corpora lutea. On day 0, cows received a second dosage of GnRH (**GnRH2**) to induce a preovulatory gonadotropin surge. On day 1, approximately 19 h after GnRH2 administration, each cow’s largest follicle underwent transvaginal aspiration by one of four experienced technicians to collect the preovulatory follicle contents (Moorey et al., 2021; Read et al., 2021b).

### Transvaginal aspiration of the preovulatory follicle

We performed transvaginal aspiration of the preovulatory follicle to collect oocyte and follicular fluid samples approximately 19 h after GnRH2 administration to induce the preovulatory gonadotropin surge. We aimed to determine the functional significance of variations in preovulatory follicle maturity at the time of GnRH2 administration on follicular fluid metabolome profiles and oocyte metabolic capacity after progression toward oocyte maturation. Prior to aspiration, all cows received a spinal block via administration of lidocaine (2% lidocaine, 5 mL) into the spinal cord at the first intercoccygeal space of the tailhead. The perineal area of each cow was then cleaned of all contaminants and an ultrasound-guided aspiration device attached to a Samsung HM70A ultrasound and CF4-9 convex probe was inserted into the anterior vagina. The ultrasound device consisted of an 18-gauge needle and a series of tubing to facilitate the removal of follicular contents. The ovary containing the preovulatory follicle was located and positioned for follicle aspiration before the needle was gently pushed through the vaginal wall and guided through the ovarian cortex into the antrum of the preovulatory follicle. Follicular fluid was withdrawn into a clean 12 mL syringe before the syringe was removed, replaced, and the follicle lavaged with PVA TL-HEPES (114.0 mM NaCl, 3.2 mM KCl, 2.0 mM NaHCO_3_, 0.34 mM NaH_2_PO_4_, 0.50 μM MgCl2*6H_2_O, 10.0 mM HEPES, 2.0 mM CaCl_2_*H_2_O, 2.3 μM PVA, and 12 mM sorbitol) multiple times to collect remaining follicular cells.

### Detection of estrus, ovarian mapping, and determination of cow weight and body condition score

Patches for the detection of estrus (Estrotect; Rockway Inc; Spring Valley, WI, USA) were placed on the tailhead of all cows at the time of PGF administration during presynchronization and on day −9 of synchronization. Patches were visually assessed 66 h after presynchronization PGF administration to determine the expression of estrus during presynchronization and on days −2 and 0 of synchronization to detect any animals that displayed estrus between GnRH1 and PGF (*n* = 0) or between PGF and follicle aspiration (removed from the study, *n* = 5). Patches were scored on a scale of 0 to 4 with 0 being a missing patch, and 1 to 4 equating to <25% rubbed (patch = 1), 25% to 50% rubbed (patch = 2), 50% to 75% rubbed (patch = 3), and >75% rubbed (patch = 4). Estrus was recorded if patch score equaled 3 or 4.

On days −9, −2, 0, and 1, ovaries of all cattle were examined by an experienced technician using transrectal ultrasonography with a Samsung HM70A ultrasound and CF4-9 convex probe. All follicles >7 mm in diameter and all corpora lutea were recorded. Follicle size was calculated for all recorded follicles by averaging the measures of the largest diameter and the diameter perpendicular to it. Body weights of all animals were collected and body condition score (Whitman, 1975; scale of 1, emaciated to 9, obese) was assigned on day 1.

### Blood collection and processing

Blood samples were collected on days −9, −2, 0, and 1. Approximately 10 mL of blood was collected via the tailvein using vacutainers for serum collection and 18-gauge, 1-inch long needles (Becton, Dickinson and Company; Franklin Lakes, NJ, USA). Blood was allowed to clot for at least 1 h before being refrigerated at 4 °C for approximately 24 h. Samples were then centrifuged at 1,200 × *g* for 25 min at 4 °C and the serum supernatant was collected into borosilicate glass tubes and stored at −20 °C until processing for estradiol and progesterone hormone assays (VWR, Radnor, PA, USA). Samples collected in year 1 were stored for 19 mo and samples collected in year 2 were stored for 7 mo prior to processing.

### Processing of follicular fluid and oocyte samples

Follicular fluid and PVA TL-HEPES media flushes collected during follicle aspiration were deposited into individual wells of a four-well Petri plate and searched to find the COC. Once located, the COC was placed into a four-well plate containing 1 mL of 1X trypsin (Thermo Fisher Scientific, Waltham, MA, USA). The oocyte was denuded by gently pipetting the COC within the trypsin using a Cook Flexipet Adjustable Handle pipette with a 170 μm Cook Flexipet pipette tip (Cook Group, Bloomington, IN, USA). The oocyte underwent three consecutive washes in phosphate-buffered saline (**PBS**) before being snap-frozen in a 2 mL cryovial (Neptune Scientific, San Diego, CA, USA) containing 2 μL PBS. The follicular fluid was then collected into 1.7 mL tubes (VWR) and centrifuged at 4 °C for 5 min at 500 × *g* to remove the remaining cellular debris. The follicular fluid supernatant was distributed between a borosilicate glass tube and 2 mL cryovials. Follicular fluid samples in glass tubes were stored at −20 °C, and samples stored in cryovials were snap-frozen in liquid nitrogen for storage at −80 °C until further processing.

### Oocyte ATP and mtDNA copy number quantification

Quantification of oocyte ATP levels and mtDNA copy number were performed in single oocytes according to a protocol previously optimized by our lab (*n* = 110; Read et al., 2021a). Briefly, oocytes were lysed by adding 8 μL of 5 mM Tris HCl to each tube and heating for 10 min at 95 °C. The lysate was then divided in half with 5 μL used to quantify intra-oocyte ATP levels and the remaining 5 μL used to quantify mtDNA copy number. Quantification of ATP levels was performed in duplicate using the ATP Determination Kit according to the manufacturer’s directions (Life Technologies; Carlsbad, CA, USA). The portion of the lysate for mtDNA copy number analysis was combined with proteinase K (final concentration of 200 μg/mL; Zymo Research, Irvine, CA, USA) and heated at 55 °C to expose the mtDNA. A custom TaqMan assay was designed to determine the mtDNA copy number of each oocyte (Thermo Fisher Scientific; Read et al., 2021a). TaqMan primer and probes were combined with the oocyte lysate and Fast Advanced Master Mix (Thermo Fisher Scientific) and analyzed in duplicate using a QuantStudio3 (Thermo Fisher Scientific). The PCR settings were as follows: 2 min at 94 °C followed by 40 cycles of 10 s at 94 °C, 15 s at 57 °C, and 12 s at 72 °C. Average sample values for each assay were compared with the values from a known standard curve (ATP: 0.5, 0.05, 0.025, 0.005, and 0.0025 μM; mtDNA: 10, 100, 1,000, 10,000, 100,000, and 1,000,000 copies) and used to calculate the levels of ATP and mtDNA copy number within each oocyte. Intra-assay and inter-assay coefficient of variance (**CV**) values averaged 0.65% and 4.39% for mtDNA copy number and 13.29% and 15.16% for intraoocyte ATP.

### UHPLC-HRMS metabolomics

Follicular fluid samples corresponding to each collected oocyte (*n* = 45) from year 2 were selected for metabolomics processing. Cryovial-stored, follicular fluid samples were thawed on ice, and 50 µL aliquots of each sample were placed into individual 2 mL tubes. Each sample was analyzed by ultra-high-performance liquid chromatography–high resolution mass spectrometry (**UHPLC-HRMS**) at the University of Tennessee Biological and Small Molecule Mass Spectrometry Core (RRID: SCR_021368). Briefly, metabolites were extracted from the follicular fluid using a 20:40:40 water/methanol/acetonitrile (v/v/v) solution with 0.10 M formic acid (Martens et al., 2011; Greene et al., 2020). The metabolomes of each sample were separated on a Synergy Hydro RP (2.5 μm, 100 mm × 2.0 mm column; Phenomenex, Torrance, CA, USA) at 25 °C. The solvents for the elution were: phase A: 97:3 methanol/water (v/v) with 11 mM tributylamine and 15 mM acetic acid and phase B: 100% methanol. The solvent gradient from 0 to 5 min was 100% A:0% B, from 5 to 13 min was 80% A:20% B, from 13 to 15.5 min was 45% A:55% B, from 15.5 to 19 min was 5% A:95% B, and from 19 to 25 min was 100% A:0% B with a flow rate of 200 μL/min. Detection of the metabolome components was accomplished using an Exactive Plus Orbitrap mass spectrometer (Thermo Fisher Scientific) fitted with an electrospray ionization probe operated in negative mode. The scan range was 72 to 1,000 *m/z*, the resolution was set to 140,000, and the acquisition gain control target to 3e6. Files were generated by the HRMS in the Xcalibur (RAW) format and were converted to the open-source mzML format via the open-source msconvert software, which is part of the ProteoWizard package (Chambers et al., 2012; Clasquin et al., 2012). MAVEN (mzroll) software, Princeton University (Melamud et al., 2010), which uses a grouping algorithm for nonlinear retention time alignment, was used to pick peaks, integrate intensities, and visualize the data and extracted ion chromatograms. Preprocessed data from MAVEN was used to conduct all further biological and statistical analyses.

### Quantification of estradiol and progesterone concentrations in serum and follicular fluid

Serum collected on days −2, 0, and 1 (PGF, GnRH2, and follicle aspiration timepoints, respectively) that corresponded to each collected oocyte (*n* = 110) underwent estradiol radioimmunoassay as previously described (Kirby et al., 1997). Intra-assay and inter-assay CV values averaged 3.49% and 10.74%. Serum collected on days −9, −2, 0, and 1 (GnRH1, PGF, GnRH2, and follicle aspiration timepoints, respectively) that corresponded to each collected oocyte (*n* = 110) as well as the follicular fluid stored in the glass tubes (*n* = 106) were analyzed for progesterone concentration using the ImmuChem progesterone double-antibody radioimmunoassay kit (MP Biomedicals; Pohler et al., 2016; Rispoli et al., 2019) per manufacturer’s instructions. Due to limitations in sample volume, three serum samples from the GnRH1 timepoint and four follicular fluid samples were not analyzed. Follicular fluid samples were diluted from 1:5 to 1:20 to ensure it was within the range of detection of the assay. Intra-assay and inter-assay CV values averaged 5.97% and 5.66% for serum samples and 4.54% and 3.52% for follicular fluid samples. The follicular fluid stored in glass tubes corresponding to the collected oocytes (*n* = 106) was also analyzed for estradiol concentration using the DetectX1 serum 17β-estradiol multispecies enzyme immunoassay (Arbor Assays, Ann Arbor, MI, USA) per manufacturer’s instructions. Samples were diluted from 1:20 to 1:2,500 to ensure they were within the detectable range of the assay. Intra-assay and inter-assay CV values were 6.67% and 8.58%.

### Statistical analyses

Before statistical analyses were performed, steroid hormone profiles in serum, follicle dynamics, and/or follicular fluid estradiol:progesterone ratios were utilized to identify cattle that underwent luteolysis following PGF administration, developed a growing preovulatory follicle, and responded to the induced preovulatory gonadotropin surge. Successful luteolysis was defined as serum progesterone <1 ng/mL at the GnRH2 and follicle aspiration timepoints (Tenhagen et al., 2000; Rispoli et al., 2019; Madureira et al., 2021). Development of a growing preovulatory follicle was classified as an increase in serum estradiol concentration and follicle diameter between PGF and GnRH2 (Alvarez et al., 2000; Fortune et al., 2004; Perry et al., 2014). Response to the administration of GnRH2 was verified by a decrease in serum estradiol between GnRH2 and follicle aspiration and follicular fluid estradiol:progesterone ratio <3 (Fortune and Hansel, 1985). A total of 30 oocytes were removed from analyses due to their corresponding hormonal profiles not meeting these criteria. All analytical procedures were performed using R software (R Core Team, 2020), and the corresponding code is available online (https://github.com/CaseyRead/Read_etal_2022_JAS). Oocytes with ATP and mtDNA values inside the standard curve and ideal hormonal profiles were used for analysis (*n* = 52). All values are presented as mean ± SD.

A mixed effects linear model with the random effect of year was used to perform intraoocyte ATP, mtDNA copy number, and ATP per mtDNA copy number analyses and identify cow trait or synchronization timeline parameter covariates. Linear regression (cow weight, cow age in days, days postpartum at aspiration, hours between PGF and GnRH2, follicular fluid progesterone, follicular fluid estradiol, and hours between GnRH2 and aspiration) or analysis of variance (serum progesterone ≥1 vs. <1 ng/mL at the start of synchronization, BCS) were used to determine any effects of potential covariates on preovulatory follicle diameter and serum estradiol at GnRH2 or oocyte ATP, oocyte mtDNA copy number, and oocyte ATP produced per mtDNA copy number. After initial analyses of each covariate, variables that were significantly related to ATP, mtDNA copy number, or ATP/mtDNA copy number were included in the respective models along with follicle diameter and serum estradiol at GnRH2. We then utilized a top-down, stepwise approach to remove covariates that lost significance (*P* > 0.10) in the full model and reach a final model for ATP, mtDNA copy number, or ATP/mtDNA copy number analyses. For ATP and ATP/mtDNA copy number analyses, the final model contained our independent variables of interest (follicle diameter and serum estradiol at GnRH2) as well as time from GnRH2 to follicle aspiration. For the mtDNA copy number analysis, the final model only contained preovulatory follicle diameter and serum estradiol concentration at GnRH2.

Linear regression and analysis of variance were also performed where appropriate to determine the effects of cow trait and/or synchronization timeline parameters on the 90 individual follicular fluid metabolites. A similar top-down, stepwise approach was taken to determine the best model for each individual metabolite. Each cow trait and timeline parameter was included in the full model and any covariates with *P* > 0.05 were removed. Once the final model was obtained for each metabolite, the false discovery rate (**FDR**) was calculated for the *P*-values of preovulatory follicle diameter, serum estradiol concentration at GnRH2, and each covariate. Metabolites were considered significantly related to each predictive variable if FDR < 0.10 was observed. Metaboanalyst 5.0 (Pang et al., 2021) was used to perform Kyoto Encyclopedia of Genes and Genomes (**KEGG**) pathway enrichment analysis of metabolites whose abundance was correlated with variables of interest or covariates. Enrichment of pathways was determined to be significant if FDR was <0.10.

The effects of individual follicular fluid metabolite on intraoocyte ATP levels were determined using linear regression with the covariates of serum estradiol at GnRH2 and hours from GnRH2 to follicle aspiration included in the model. After the FDR was calculated, no follicular fluid metabolites were found to be significant to intraoocyte ATP (FDR > 0.10), so no pathway analyses were performed.

## Results and Discussion

### Overview of animal data

All samples were collected from suckled, postpartum beef cows managed as part of the productive herd at one of the University of Tennessee AgResearch and Education Centers. Age (2,068.3 ± 648.02 d, 5.2 ± 2.3 yr), BCS (5.96 ± 1.10), and weight (617.40 ± 103.19 kg) of the 52 cattle included in our analyses were similar to previously reported values for beef cattle operations (Cushman et al., 2007, 2013; Damiran et al., 2018; Bonacker et al., 2020). To maintain the yearly breeding season schedule, animals were 58.36 ± 4.12 d postpartum at the time of preovulatory follicle aspiration. There was no effect of days postpartum or serum progesterone concentration ≥1 ng/mL vs. <1 ng/mL at the onset of synchronization on preovulatory follicle diameter, serum estradiol concentration, oocyte ATP, oocyte ATP per mtDNA copy number, or oocyte mtDNA copy number (*P* > 0.10). Largest follicle diameter at GnRH2 was 12.33 ± 1.53 mm and serum estradiol concentration at the time of GnRH2 administration was 7.23 ± 3.69 pg/mL. These measures are consistent with previously published studies investigating preovulatory follicle diameter or estradiol production (Geary et al., 2001; Lamb et al., 2001; Perry et al., 2005, 2007; MacNeil et al., 2006; Atkins et al., 2013; Moorey et al., 2021; Read et al., 2021b). Serum estradiol and follicle diameter at GnRH2 were not associated with cow phenotypic traits of age, BCS, weight, and days postpartum or synchronization timeline parameters of time between GnRH1 and PGF, PGF and GnRH2, or GnRH2 and follicle aspiration (*P* > 0.05).

Oocytes and follicular fluid samples utilized in this study were collected via transvaginal follicle aspiration of the preovulatory follicle 19.02 ± 1.90 h after administration of GnRH2. Follicular fluid progesterone and estradiol concentrations averaged 68.57 ± 46.88 ng/mL and 52.02 ± 48.75 pg/mL, respectively, and were positively related to each other (*P* = 0.0009). At this time, the induced luteinizing hormone (**LH**) surge should have stimulated the follicle’s progression toward ovulation resulting in alterations in the preovulatory follicular environment. At this timepoint after GnRH administration, the somatic follicular cells have begun luteinization and are increasing their production of progesterone, cumulus cells are expanding, and the oocyte has achieved metaphase I (**MI**) and has reached or is progressing towards metaphase II (**MII**; Hyttel et al., 1986, 1997; de Loos et al., 1991; Richards et al., 1998; Iwata et al., 2011; Pulley et al., 2015). The mitochondria within oocytes of this maturation stage would have increased their production of ATP in order to support progression through meiotic maturation (Fair et al., 1997; Jeong et al., 2009; Dalbies-Tran et al., 2020). Measuring the metabolic capacity of the oocyte at this time point should reveal differences in the acquisition of metabolic competence of oocytes within follicles of varied physiological maturity when exposed to an induced preovulatory gonadotropin surge. Alterations in the metabolome of follicular fluid samples collected at this time after premature induction of the gonadotropin surge could highlight both lasting implications of the follicle’s physiological status at the time of the surge as well as impacts of the follicle’s pre-surge developmental status on metabolic processes occurring in COC and granulosa cells.

### The impact of preovulatory follicle physiological status on oocyte metabolic capacity

Amount of ATP (pg) and mtDNA copy number were used to quantify the metabolic capacity of individual oocytes. Intraoocyte ATP ranged from 55.31 to 1,111.30 pg (532.29 ± 279.15 pg) and mtDNA copy number ranged from 25,355 to 1,070,864 copies (535,696 ± 419,745.3 copies). ATP values were higher than those previously reported by others, however, this could be due to differences in assay sensitivity, dilution factors, and/or other sample processing variations (Stojkovic et al., 2001; Tamassia et al., 2004; Jiao et al., 2007; Iwata et al., 2011; Scantland et al., 2014; Dadarwal et al., 2017; Payton et al., 2018; Hashimoto et al., 2019). The mtDNA copy number values obtained in this study were consistent with previously published literature (Tamassia et al., 2004; Jiao et al., 2007; Chiaratti et al., 2010; Iwata et al., 2011; Hashimoto et al., 2019).

**Figure 2.**
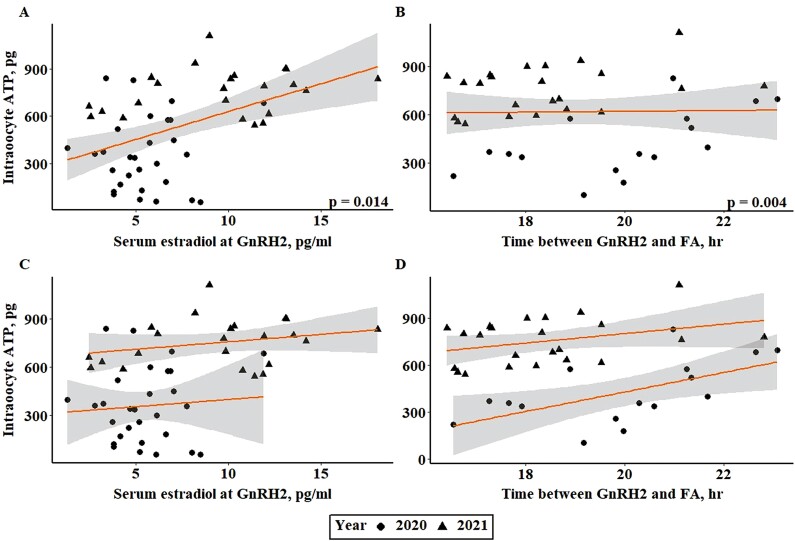
Relationship between serum estradiol concentration at GnRH2 and intraoocyte ATP (pg; A, C). Relationship between time from GnRH2 to FA and intraoocyte ATP (pg; B, D). Panels A and B depict the line of best fit for both years of the study, and panels C and D depict the line of best fit for each year individually. GnRH2, gonadotropin-releasing hormone administration to induce the preovulatory gonadotropin surge; FA, preovulatory follicle aspiration.

**Figure 3.**
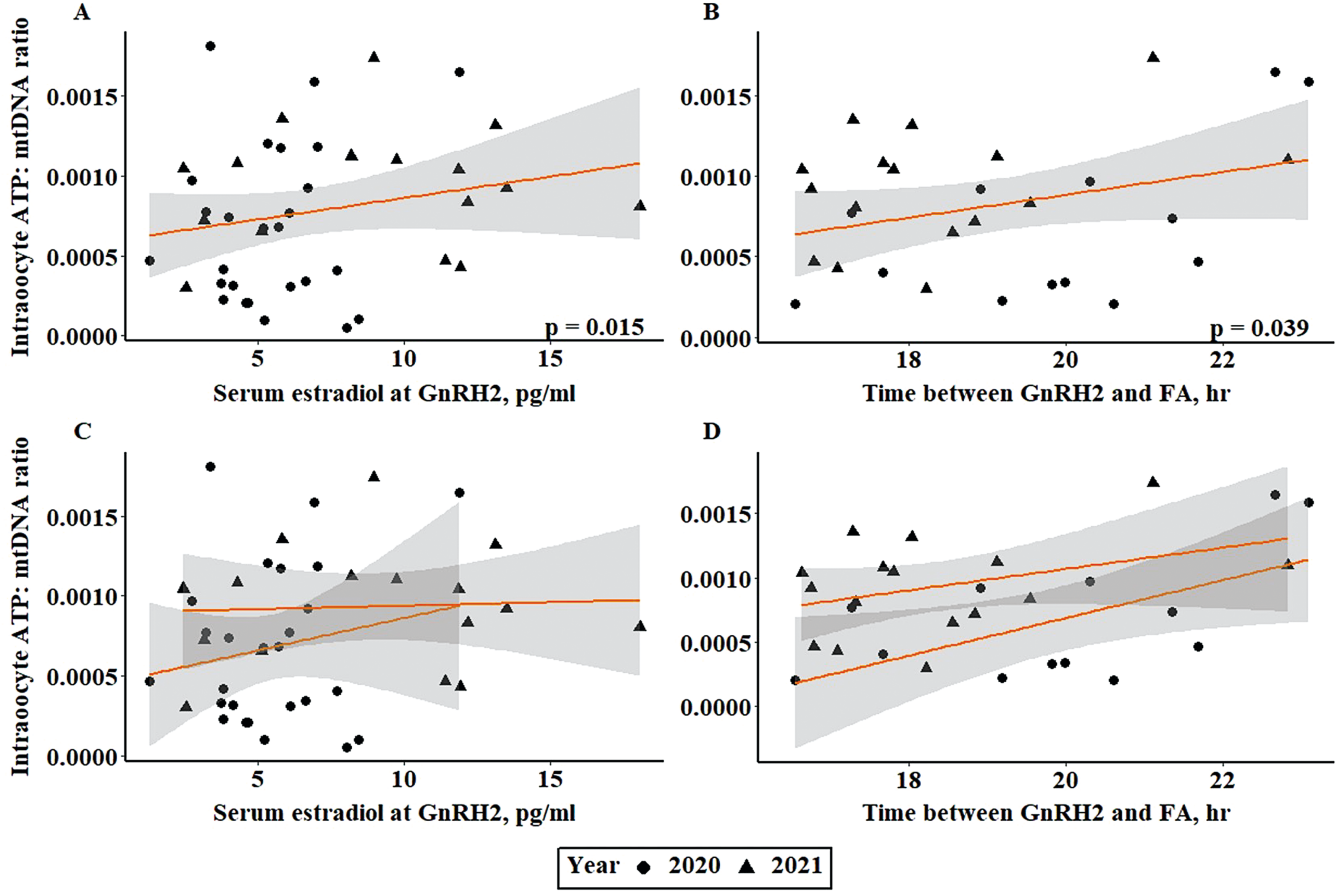
Relationship between serum estradiol concentration at GnRH2 and intraoocyte ATP per mtDNA copy number ratio (A, C). Relationship between time from GnRH2 to FA and intraoocyte ATP per mtDNA copy number ratio (B, D). Panels A and B depict the line of best fit for both years of the study, and panels C and D depict the line of best fit for each year individually. GnRH2, gonadotropin-releasing hormone administration to induce the preovulatory gonadotropin surge; FA, preovulatory follicle aspiration; mtDNA, mitochondrial DNA copy number.

Intraoocyte ATP at approximately 19 h after GnRH2 administration was related to serum estradiol concentration at GnRH2 (*P* = 0.014; Figure 2A) and time between GnRH2 and follicle aspiration (*P* = 0.004; Figure 2B). Follicle diameter at GnRH2, however, was not related to oocyte ATP level approximately 19 h after GnRH administration (*P* = 0.085). Though samples collected from year 2 had higher levels of both intraoocyte ATP and serum estradiol concentration (*P* < 0.001), similar trends for the relationship between oocyte ATP level and serum estradiol concentration or time from GnRH2 to follicle aspiration were observed in each year (Figure 2C and D). mtDNA copy number was not related to follicle diameter at GnRH2, serum estradiol concentration at GnRH2, or any potential covariates (*P* > 0.10). The lack of relationship between mtDNA copy number and physiological status of the follicle and/or intraoocyte ATP is not surprising. There is variation in the published literature on the relationship between mtDNA copy number and oocyte developmental competence (Reynier et al., 2001; Tamassia et al., 2004; May-Panloup et al., 2007; Wai et al., 2010; Iwata et al., 2011; Hashimoto et al., 2019). A study by van Blerkom et al. (2004) suggested that mtDNA copy number could be linked to oocyte developmental competence if the metabolic capacity of the oocyte was accounted for. Therefore, we performed an additional analysis to determine if there was an impact of follicle physiological status on the amount of intraoocyte ATP present per mtDNA copy number. The amount of ATP produced per mtDNA copy number was related to serum estradiol concentration at GnRH2 (*P* = 0.015; Figure 3A and C) and time from GnRH2 to follicle aspiration (*P* = 0.039; Figure 3B and D).

Increased level of oocyte ATP has been associated with increased developmental competence of the oocyte (Quinn and Wales, 1973; Calarco, 1995; Stojkovic et al., 2001; Tamassia et al., 2004; Dalton et al., 2014). Therefore, the positive relationship between serum estradiol concentration at GnRH2 and both oocyte ATP level and oocyte ATP production per mitochondria likely contributes to the positive relationship between serum estradiol concentration at GnRH2 and day 7 embryo cleavage and quality grades previously observed by Atkins et al. (2013). As the dominant follicle progresses through proestrus, it grows in diameter and produces increasing levels of estradiol (Alvarez et al., 2000; Fortune et al., 2004; Perry et al., 2005, 2014). Though both preovulatory follicle diameter and serum estradiol concentration have been previously associated with embryo cleavage, embryo quality, and pregnancy success in animals that did not exhibit estrus, data from animals that did exhibit estrus and experience an endogenous gonadotropin surge determined that preovulatory follicle diameter did not influence pregnancy outcome in estrual cows (Perry et al., 2005; Atkins et al., 2013). Therefore, preovulatory follicle diameter is primarily an indicator of the follicle’s physiological maturity and its proximity to reaching estradiol production required to stimulate estrus and an endogenous gonadotropin surge. As such, there is variation in serum estradiol concentration across preovulatory follicle diameters among cows requiring exogenous GnRH to induce ovulation (Jinks et al., 2013), which indicates that follicle maturity is likely equally variable across such follicles. The results of the present study support the notion that serum estradiol concentration is more closely tied to follicle maturity prior to the induced gonadotropin surge than follicle diameter and may have a stronger correlation with oocyte metabolic capacity. Though previous in vitro studies using oocytes collected from antral follicles from ~2 to 13 mm in diameter have reported positive relationships between follicle size and oocyte developmental competence (Pavlok et al., 1992; Lonergan et al., 1994; Blondin and Sirard, 1995; Hagemann et al., 1999), this study represents a key increase in knowledge because it provides the basis for increased oocyte metabolic competency based on follicle status directly before the preovulatory gonadotropin surge. Though in vivo oocyte maturation has been regarded as a “gold standard” for oocyte developmental competence (van de Leemput et al., 1999; Dieleman et al., 2002), this study demonstrates clear differences in the metabolism of follicular cells of preovulatory follicles that were of varied degrees for maturity before the onset of in vivo oocyte maturation.

The positive relationship between intraoocyte ATP (pg) at approximately 19 h post GnRH2 and serum estradiol concentration at the time of GnRH2 likely indicates that the oocyte was allowed to progress further through capacitation prior to induction of the LH surge and oocyte maturation. The oocyte would have an improved ability to produce ATP due to an increased opportunity to accumulate the stores of metabolic substrates like pyruvate, lactate, and lipids through gap junctional transfer from the cumulus cells prior to maturation and the disruption of such transfer (Fair et al., 1997; Swain and Pool, 2008; Zhang and Smith, 2015; Reader et al., 2017; Conti and Franciosi, 2018). These metabolic substrates are required by the oocyte or early embryo’s mitochondria to produce ATP during oocyte maturation and embryo development to the blastocyst stage (Biggers et al., 1967; Chappel, 2013; Ge et al., 2013; Dalton et al., 2014; Aguila et al., 2020). Aside from indicating advanced follicle maturity prior to the induced gonadotropin surge, increased estradiol concentration at GnRH2 may also have a more direct effect on COC metabolism as exposure of the COC to estrogen increases the expression of gap junctional proteins and increases their localization to the plasma membrane (Firestone and Kapadia, 2012). This increase in functional gap junctions enhances the ability of the cumulus cells to transport cGMP to the oocyte as well as the metabolic substrates needed to support oocyte and embryo development. Because the oocyte relies on gap junction transport to store pyruvate, lipids, and other metabolic substrates, increased functional gap junctions during proestrus could allow for more effective transport of these materials and thus greater ATP levels in oocytes collected from preovulatory follicles of increased estradiol production.

As the oocyte progresses from the germinal vesicle (**GV**) stage to MII, the activity of its mitochondria increases to produce the ATP necessary to sustain progression through meiotic maturation (Fair et al., 1997; Jeong et al., 2009; Chappel, 2013; Dalton et al., 2014; Aguila et al., 2020). The positive relationship between time from GnRH2 to follicle aspiration and ATP level or ATP per mtDNA copy number observed in the present study reflects this increase in mitochondrial activity during oocyte maturation. Because the preovulatory gonadotropin surge, and thus the onset of oocyte maturation, is induced approximately 2 h after GnRH is administered (Giordano et al., 2012), the positive correlation between the time from GnRH2 to follicle aspiration is also related to the stage of meiotic resumption of the oocyte. Oocytes in the current study were collected from 16.42 to 23.08 h after GnRH2 administration and, thus, would have reached approximately 15 to 21 h of oocyte maturation. In vitro studies have indicated that bovine oocytes reach MI at approximately 10 to 15 h of maturation and MII at approximately 18 to 24 h of maturation (Sirard et al., 1989; Fair et al., 1995; Hyttel et al., 1997). Therefore, the oocytes utilized in this study should all have completed MI and have reached or are nearing MII. Multiple studies have demonstrated that intraoocyte ATP increases as oocytes progress from GV to MII (Jiao et al., 2007; Iwata et al., 2011; Read et al., 2021a). Therefore, increased time between the administration of GnRH2 and follicle aspiration would result in an oocyte closer to MII with increased levels of intraoocyte ATP.

### The impact of preovulatory follicle physiological status on follicular fluid metabolites

**Figure 4.**
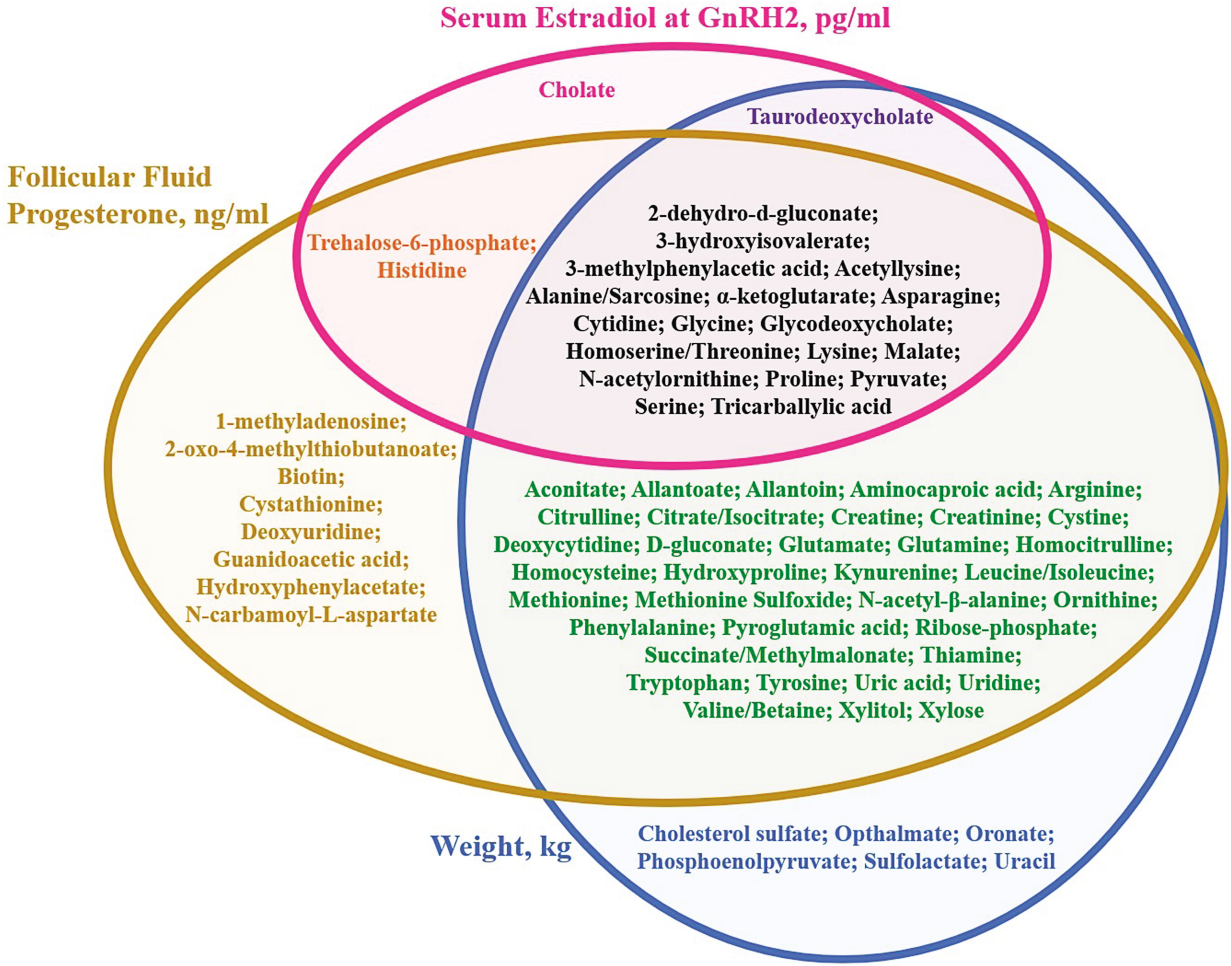
Venn diagram listing the follicular fluid metabolites whose abundance in follicular fluid collected approximately 19 h after GnRH2 administration was significantly related to serum estradiol concentration at GnRH2 (pg/mL), follicular fluid progesterone concentration at follicle aspiration (ng/mL), and cow weight (kg). GnRH2, gonadotropin-releasing hormone administration to induce the preovulatory gonadotropin surge.

We previously determined that preovulatory follicle diameter at GnRH2 was positively correlated with the abundance of follicular fluid metabolites involved in glucose metabolism, proteinogenesis, DNA methylation, and oxidative balance (Read et al., 2021b). In the current study, we aimed to determine the repeatability of such a relationship and determine if the abundance of follicular fluid metabolites was related to intraoocyte ATP levels. We detected 90 metabolites in the follicular fluid of the aspirated preovulatory follicles (Supplementary Table S1). Serum estradiol concentration at GnRH2 was positively associated with the abundance of 22 follicular fluid metabolites (FDR < 0.10; Figure 4, Supplementary Table S1). There was no relationship between follicle diameter at GnRH2 and abundance of any metabolites (FDR > 0.90; Supplementary Table S1). As discussed earlier regarding the relationship between oocyte ATP level and both serum estradiol concentration and follicle diameter at GnRH2, it is likely that serum estradiol concentration was a stronger indicator of follicle maturity prior to GnRH2 administration than follicle size. Our previous study did not include hormone profiles in serum or follicular fluid in its analyses (Read et al., 2021b). This discrepancy likely explains some of the lack of consistency between the current and previous studies in regard to the relationship between preovulatory follicle diameter and the abundance of follicular fluid metabolites. However, four of the metabolites positively related to serum estradiol at GnRH2 were previously determined to be positively correlated with follicle diameter at GnRH2 (2-dehydro-d-gluconate, 3-methylphenylacetic acid, α-ketoglutarate, and pyruvate; Read et al., 2021b). It is extremely interesting that of these four metabolites, 2-dehydro-d-gluconate, α-ketoglutarate, and pyruvate are involved in or byproducts of glucose metabolism, which appears to be a key deficiency in COCs from small or physiologically immature follicles that are prematurely exposed to the gonadotropin surge (Moorey et al., 2021; Read et al., 2021b).

Follicular fluid progesterone concentration at the time of follicle aspiration was associated with the abundance of 62 metabolites (FDR < 0.10; Figure 4, Supplementary Table S1). A positive relationship between follicular fluid progesterone concentration and metabolite abundance was identified in each significant metabolite with the exception of N-carbamoyl-l-aspartate and aspartate. Follicular fluid estradiol concentration at the time of follicle aspiration was significantly related to the abundance of only three follicular fluid metabolites (FDR < 0.10; Supplementary Table S1). The lack of relationship between many follicular fluid metabolites and follicular fluid estradiol concentration is likely the result of the sample collection timeline. Follicular fluid was collected at ~19 h after GnRH2 administration when the follicle was not in an estrogen active state but had transitioned to the production of progesterone. Animal weight was negatively related to the abundance of 60 metabolites and positively related to one metabolite, phosphoenolpyruvate (FDR < 0.10; Figure 4, Supplementary Table S1). Covariates of BCS, hours from GnRH2 to follicle aspiration, and/or days postpartum also contributed to the abundance of 11 follicular fluid metabolites (FDR < 0.10; Supplementary Table S1).

**Figure 5.**
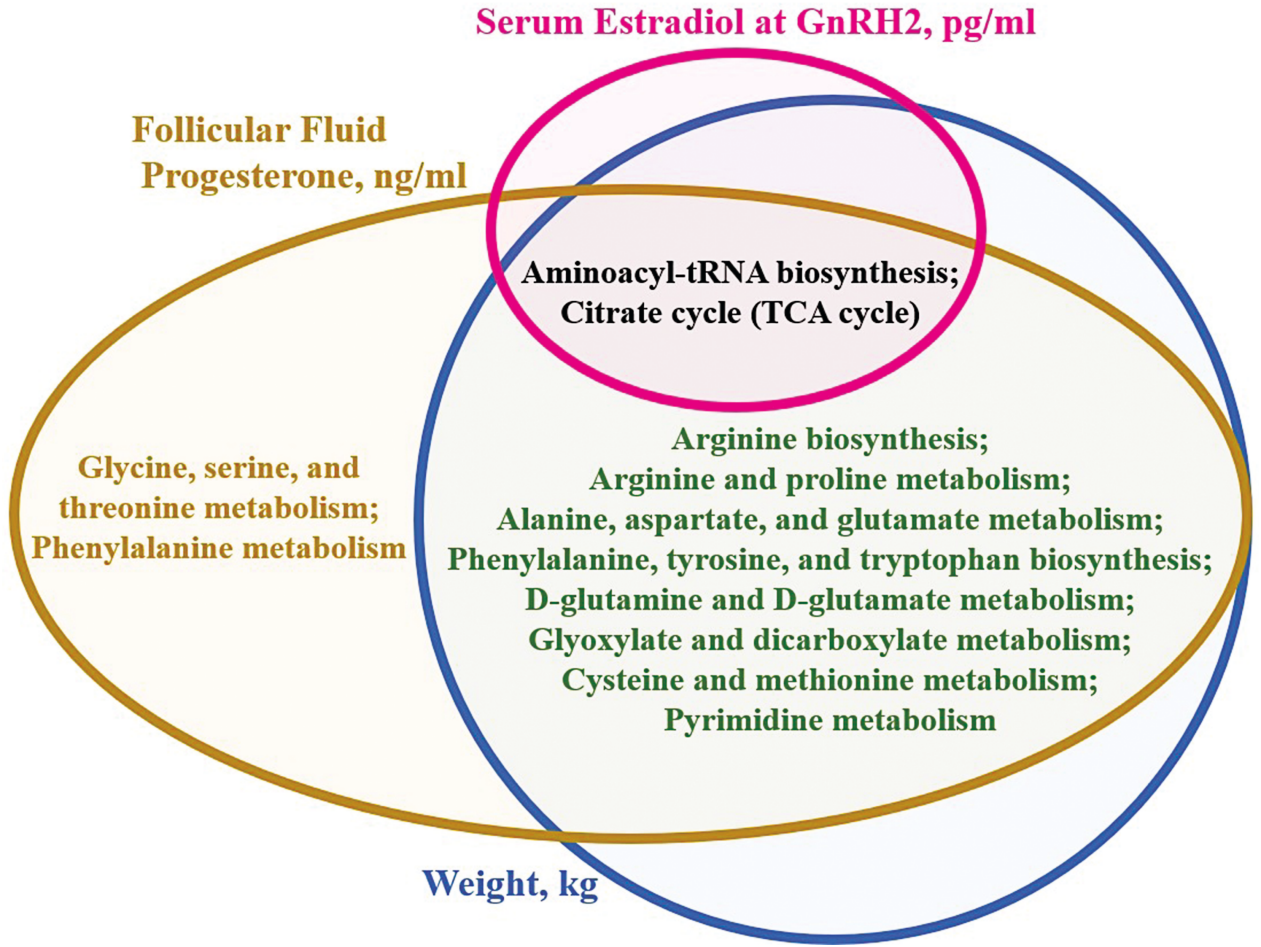
Venn diagram listing the KEGG pathways significantly enriched with follicular fluid metabolites related to serum estradiol concentration at GnRH2 (pg/mL), follicular fluid progesterone concentration at follicle aspiration (ng/mL), and cow weight (kg). GnRH2, gonadotropin-releasing hormone administration to induce the preovulatory gonadotropin surge.

The KEGG pathways “Aminoacyl tRNA biosynthesis” and the “Citrate cycle” were enriched with metabolites positively related to serum estradiol concentration at GnRH2 (FDR < 0.10; Figure 5, Supplementary Table S2). Interestingly, metabolites associated with follicular fluid progesterone concentration at follicle aspiration and with cow weight also were enriched within KEGG pathways “Aminoacyl tRNA biosynthesis” and “Citrate cycle” as well as “Arginine biosynthesis,” “Arginine and proline metabolism,” “Alanine, aspartate, and glutamate metabolism,” “d-glutamine and d-glutamate metabolism,” “Glyoxylate and dicarboxylate metabolism,” “Cysteine and methionine metabolism,” “Phenylalanine, tyrosine, and tryptophan biosynthesis,” and “Pyrimidine metabolism” (FDR < 0.10; Figure 5, Supplementary Tables S3 and S4). The KEGG pathways “Glycine, serine, and threonine metabolism” and “Phenylalanine metabolism” were also enriched with metabolites related to follicular fluid progesterone concentration at follicle aspiration (FDR < 0.10; Figure 5, Supplementary Table S3).

Metabolites that were associated with serum estradiol concentration at GnRH2, follicular fluid progesterone concentration at follicle aspiration, and/or weight were primarily involved in proteinogenesis, the citric acid cycle, and pyrimidine metabolism. Follicular fluid composition fluctuates throughout the estrous cycle and can be influenced by multiple extraovarian factors including ovarian stimulation protocols (Fortune and Hansel, 1985; Assey et al., 1994; Ambrose et al., 2006; Leroy et al., 2006, 2011; Bender et al., 2010; Von Wald et al., 2010; Wonnacott et al., 2010; Zachut et al., 2010; Liu et al., 2012; Alves et al., 2014; O’Doherty et al., 2014; Palini et al., 2014; Moreno et al., 2015; Forde et al., 2016; Takeo et al., 2017). Inducing the preovulatory gonadotropin surge in physiologically immature dominant follicles prior to the onset of estrus could result in decreased levels of metabolites involved in proteinogenesis and the citric acid cycle. This is consistent with our previous study that reported decreased abundance of metabolites enriched in the KEGG pathway “aminoacyl tRNA biosynthesis” as well as multiple pathways associated with the production of citric acid cycle intermediates in preovulatory follicles with decreased diameter (Read et al., 2021b). The positive relationship between serum estradiol concentration at GnRH2 administration and metabolites involved in proteinogenesis or the citric acid cycle suggests altered protein production and metabolism throughout the time period of oocyte maturation in less physiologically mature follicles near the time of the preovulatory gonadotropin surge and could contribute to reduced ATP abundance and lower developmental competence of oocytes from such follicles.

Follicular fluid collected from ovulatory follicles contains proteins that are important for luteinization, maturation of the oocyte, and expansion of the cumulus cells (Krisher, 2013; Brown et al., 2017; Ferrazza et al., 2017). Bovine ovulatory follicles have increased expression of proteins involved in inflammatory, nitric oxide synthase, and reactive oxygen species pathways compared with follicular fluid obtained from follicles prior to the LH surge (Ferrazza et al., 2017). Free amino acids in the follicular fluid are important to support the increased protein production occurring during the peri-ovulatory time period. In fact, aberrant follicular fluid protein expression is associated with reduced fertility in dairy cattle (Zachut et al., 2016). Amino acids can also be converted to substrates for the citric acid cycle (Guda et al., 2007; Martínez-Reyes and Chandel, 2020). The citric acid cycle generates ATP for cellular energy and NADH/FADH_2_ that are used during oxidative phosphorylation to generate additional energy (Fernie et al., 2004; Martínez-Reyes and Chandel, 2020). Increased levels of the metabolic substrates utilized by the citric acid cycle indicate increased citric acid cycle activity of the follicular cells. This aligns with previous studies demonstrating that cumulus cells have high glycolytic activity and NADH production (Biggers et al., 1967; Sutton-McDowall et al., 2010; Cinco et al., 2016; Richani et al., 2021).

The metabolites within the numerous pathways that were associated with follicular fluid progesterone or weight are primarily involved in the citric acid cycle. They are either components of the citric acid cycle (pyruvate, malate, α-ketoglutarate, and cis-aconitate) or are amino acids that can be converted into cycle intermediates (tyrosine, phenylalanine, asparagine, glycine, cysteine, tryptophan, arginine, histidine, glutamine, glutamate, and methionine). There may be a direct effect of follicular fluid progesterone concentration at the time of follicle aspiration on both COC metabolism and follicular fluid metabolites. Both bovine cumulus cells and oocytes express progesterone receptors (Aparicio et al., 2011). In somatic cells, progesterone increases oxidative phosphorylation and beta-oxidation (Behera et al., 2009; Dai et al., 2019, 2020; Lee et al., 2021). During oocyte meiotic maturation, energy production via oxidative phosphorylation increases (Biggers et al., 1967; Babayev and Seli, 2015; Coticchio et al., 2015; Brown et al., 2017). Beta-oxidation of fatty acids also increases and is used to generate substrates for use in the citric acid cycle (Hyttel et al., 1986; Sturmey et al., 2009; Paczkowski et al., 2013; Dunning et al., 2014; Dalbies-Tran et al., 2020). The citric acid cycle produces ATP as well as the NADH and FADH_2_ that are required for oxidative phosphorylation (Martínez-Reyes and Chandel, 2020). Based on this information, the increased presence of citric acid cycle components in follicular fluid with increased progesterone concentration could be directly due to progesterone receptor signaling to increase oxidative phosphorylation and beta-oxidation within the oocyte and/or cumulus cells. Progesterone receptor signaling also induces the expression of genes involved with ovulation in granulosa cells (Robker et al., 2009).

Once the FDR was applied to account for testing multiple hypotheses, we did not detect a significant relationship between any follicular fluid metabolites and oocyte ATP level (FDR > 0.10). This preliminary experiment may have been performed too late in oocyte maturation to detect significant relationships among follicular fluid metabolites and oocyte ATP. Samples in the present study were collected near ovulation and the completion of oocyte maturation when the oocytes have decoupled from their surrounding cumulus cells. The collection of samples earlier or at the time of the preovulatory gonadotropin surge may have better represented the metabolic environment available to the COC before or early in maturation when a higher level of active transfer of metabolites from the cumulus cells to the oocyte was present. Potential influences of granulosa cell metabolism on the follicular fluid metabolome may have also overshadowed potential relationships between the follicular fluid metabolome and oocyte ATP at approximately 19 h after GnRH2 administration. We are optimistic to further explore relationships between the follicular fluid milieu and oocyte metabolic competency in future studies.

## Conclusions

The results of the present study provided an essential advancement to our knowledge of the relationship between preovulatory follicle physiological status near the time of an induced gonadotropin surge on oocyte developmental and metabolic competency. We identified a positive relationship between serum estradiol concentration at GnRH2 and time from GnRH2 to follicle aspiration on the availability of ATP within the oocyte after approximately 17 h of maturation. Preovulatory serum estradiol concentration’s positive association with total ATP levels and ATP produced per mtDNA copy number in oocytes may be both correlatives due to the relationship of estradiol with follicle maturity at the time of GnRH2 and causative due to the upregulation of estradiol of gap junction transfer of metabolites between the cumulus cells and oocyte. Oocyte meiotic maturation and early embryo development are energy-demanding processes, and the decreased developmental competence of oocytes following premature induction of the preovulatory gonadotropin surge in physiologically immature preovulatory follicle is likely due to decreased metabolic capacity of the oocyte. Additionally, we found a positive relationship between the physiological status of the follicle before and after exposure to the preovulatory gonadotropin surge on the metabolome of the follicular fluid. Premature exposure of the follicle to the LH surge coupled with reduced follicular fluid progesterone production post surge resulted in an altered follicular environment that could have implications for oocyte developmental competence related to metabolism and areas beyond what was measured in this study. Future studies will seek to further elucidate the effects of premature induction of ovulation on oocyte developmental competence.

## Supplementary Material

skac136_suppl_Supplementary_Tables

## Abbreviations

ATP: adenosine triphosphate
CIDR: controlled internal drug release
CL: corpus luteum
COC: cumulus–oocyte complex
DNA: deoxyribonucleic acid
ESI: electrospray ionization
FA: follicle aspiration
FADH_2_: flavin adenine dinucleotide
FDR: false discovery rate
FTAI: fixed-time artificial insemination
GnRH: gonadotropin-releasing hormone
GnRH1: the first administration of GnRH during the synchronization protocol
GnRH2: the second administration of GnRH during the synchronization protocol
GV: germinal vesicle
KEGG: Kyoto encyclopedia of genes and genomes
LH: luteinizing hormone
MI: metaphase I
MII: metaphase II
mtDNA: mitochondrial DNA
NADH: nicotinamide adenine dinucleotide + hydrogen
NOS: nitric oxide synthase
PBS: phosphate-buffered saline
PGF: prostaglandin F_2α_
ROS: reactive oxygen species
UHPLC-HRMS: ultra-high-performance liquid chromatography–high resolution mass spectrometry
