# Supplementary material for: Preovulatory serum estradiol concentration is positively associated with oocyte ATP and follicular fluid metabolite abundance in lactating beef cattle
Source: J Anim Sci. 2022 Jul 1;100(7):skac136. doi: 10.1093/jas/skac136 (PMC9246671; doi:10.1093/jas/skac136)
Supplement: skac136_suppl_Supplementary_Tables [file skac136_suppl_supplementary_tables.docx]

**Supplemental Table 1.** False discovery rate values for the relationship between follicular fluid metabolites and serum estradiol at GnRH2, pre-ovulatory follicle diameter at GnRH2, or final model covariates.

| **Metabolite** | **KEGG ID^1^** | **False Discovery Rate** | | | | | | | |
| --- | --- | --- | --- | --- | --- | --- | --- | --- | --- |
|  |  | **Serum E2^2^ at GnRH2^3^, pg/ml** | **Follicle diameter at GnRH2, mm** | **FF^4^ P4^5^ at FA^6^, ng/ml** | **Weight, kg** | **Hours GnRH2 to FA** | **BCS^7^** | **FF E2 at FA, ng/ml** | **Days postpartum** |
| taurodeoxycholate | C05463 | 0.034 | 0.998 | NA ⁵ | 0.002 | NA | NA | NA | NA |
| glycine | C00037 | 0.036 | 0.998 | 0.001 | 0.003 | NA | NA | NA | NA |
| 3-hydroxyisovalerate | C20827 | 0.038 | 0.998 | 0.002 | 0.017 | 0.046 | NA | 0.035 | NA |
| 2-dehydro-d-gluconate | C03342 | 0.045 | 0.998 | 0.005 | 0.004 | NA | NA | NA | NA |
| 3-methylphenylacetic acid | 12121^9^ | 0.045 | 0.998 | 0.006 | 0.007 | NA | NA | NA | NA |
| acetyllysine | C12989 | 0.045 | 0.998 | 0.001 | 0.003 | NA | NA | NA | NA |
| cholate | C00695 | 0.045 | 0.998 | NA | NA | NA | 0.037 | NA | NA |
| glycodeoxycholate | C05464 | 0.045 | 0.998 | 0.046 | 0.013 | NA | NA | NA | NA |
| histidine | C00135 | 0.045 | 0.998 | 0.002 | 0.129 | NA | NA | NA | NA |
| malate | C00149 | 0.045 | 0.998 | 0.005 | 0.014 | NA | NA | NA | NA |
| trehalose-6-phosphate | C00689 | 0.045 | 0.998 | 0.001 | NA | NA | NA | 0.035 | NA |
| alanine_sarcosine | C00041;  C00213 | 0.054 | 0.998 | 0.001 | 0.002 | NA | NA | NA | NA |
| alpha-ketoglutarate | C00026 | 0.054 | 0.998 | 0.003 | 0.012 | NA | NA | NA | NA |
| tricarballylic acid | C19806 | 0.054 | 0.998 | 0.015 | 0.013 | NA | NA | NA | NA |
| pyruvate | C00022 | 0.060 | 0.998 | 0.006 | 0.007 | NA | NA | NA | NA |
| asparagine | C00152 | 0.060 | 0.998 | 0.001 | 0.004 | NA | NA | NA | NA |
| cytidine | C00475 | 0.060 | 0.998 | 0.003 | 0.004 | NA | NA | NA | NA |
| proline | C00148 | 0.069 | 0.998 | 0.002 | 0.003 | NA | NA | NA | NA |
| homoserine_threonine | C00263;  C00188 | 0.075 | 0.998 | 0.002 | 0.003 | NA | NA | NA | NA |
| lysine | C00047 | 0.094 | 0.998 | 0.001 | 0.008 | NA | NA | NA | NA |
| n-acetylornithine | C00437 | 0.094 | 0.998 | 0.002 | 0.002 | NA | NA | NA | NA |
| serine | C00716 | 0.094 | 0.998 | 0.002 | 0.003 | NA | NA | NA | NA |
| ribose-phosphate | C00117 | 0.101 | 0.998 | 0.005 | 0.013 | NA | NA | NA | NA |
| allantoate | C00499 | 0.101 | 0.998 | 0.006 | 0.008 | NA | NA | NA | NA |
| citrate_isocitrate | C00158;  C00311 | 0.108 | 0.998 | 0.003 | 0.015 | NA | NA | NA | NA |
| creatinine | C00791 | 0.108 | 0.998 | 0.001 | 0.011 | NA | NA | NA | NA |
| xylose | C00181 | 0.108 | 0.998 | 0.002 | 0.004 | NA | NA | NA | NA |
| creatine | C00300 | 0.132 | 0.998 | 0.002 | 0.003 | 0.056 | NA | NA | NA |
| allantoin | C01551 | 0.135 | 0.998 | 0.002 | 0.007 | NA | NA | NA | NA |
| 1-methyladenosine | C02494 | 0.138 | 0.998 | 0.015 | NA | 0.020 | 0.049 | 0.042 | NA |
| homocysteine | C00155 | 0.138 | 0.998 | 0.002 | 0.019 | NA | NA | NA | NA |

**Supplemental Table 1 continued.** False discovery rate values for the relationship between follicular fluid metabolites and serum estradiol at GnRH2, pre-ovulatory follicle diameter at GnRH2, or final model covariates.

| **Metabolite** | **KEGG ID^1^** | **False Discovery Rate** | | | | | | | |
| --- | --- | --- | --- | --- | --- | --- | --- | --- | --- |
|  |  | **Serum E2^2^ at GnRH2^3^, pg/ml** | **Follicle diameter at GnRH2, mm** | **FF^4^ P4^5^ at FA^6^, ng/ml** | **Weight, kg** | **Hours GnRH2 to FA** | **BCS^7^** | **FF E2 at FA, ng/ml** | **Days postpartum** |
| kynurenine | C00328 | 0.138 | 0.998 | 0.009 | 0.004 | NA | NA | NA | NA |
| uridine | C00299 | 0.139 | 0.998 | 0.004 | 0.017 | NA | NA | NA | NA |
| n-acetyl-beta-alanine | C01073 | 0.139 | 0.998 | 0.013 | 0.010 | NA | NA | NA | NA |
| glutamine | C00064 | 0.140 | 0.998 | 0.001 | 0.004 | NA | NA | NA | NA |
| homocitrulline | C02427 | 0.140 | 0.998 | 0.015 | 0.024 | NA | NA | NA | NA |
| succinate_methylmalonate | C00042;  C02170 | 0.140 | 0.998 | 0.023 | 0.039 | NA | NA | NA | NA |
| methionine | C00073 | 0.141 | 0.998 | 0.006 | 0.034 | NA | NA | NA | NA |
| phosphoenolpyruvate | C00074 | 0.141 | 0.998 | NA | 0.0276 | 0.056 | NA | NA | NA |
| hydroxyproline | C01157 | 0.152 | 0.998 | 0.0002 | 0.002 | NA | NA | NA | NA |
| xylitol | C00379 | 0.152 | 0.998 | 0.013 | 0.070 | NA | NA | NA | NA |
| deoxycytidine | C00881 | 0.164 | 0.998 | 0.004 | 0.011 | NA | NA | NA | NA |
| tryptophan | C00078 | 0.183 | 0.998 | 0.015 | 0.031 | NA | NA | NA | NA |
| aconitate | C00417 | 0.198 | 0.998 | 0.003 | 0.031 | NA | NA | NA | NA |
| phenylalanine | C00079 | 0.198 | 0.998 | 0.023 | 0.025 | 0.406 | NA | NA | NA |
| pyroglutamic acid | C01879 | 0.198 | 0.998 | 0.005 | 0.007 | NA | NA | NA | NA |
| tyrosine | C00082 | 0.198 | 0.998 | 0.006 | 0.019 | NA | NA | NA | NA |
| n-carbamoyl-l-aspartate | C00438 | 0.200 | 0.739 | 0.006 | NA | NA | NA | NA | NA |
| valine_betaine | C00183;  C00719 | 0.200 | 0.998 | 0.006 | 0.013 | NA | NA | NA | NA |
| d-gluconate | C00257 | 0.201 | 0.998 | 0.004 | 0.004 | NA | NA | NA | NA |
| sulfolactate | C11537 | 0.209 | 0.998 | NA | 0.031 | NA | NA | NA | NA |
| thiamine | C00378 | 0.215 | 0.998 | 0.003 | 0.008 | NA | NA | NA | NA |
| aminocaproic acid | C02378 | 0.228 | 0.998 | 0.007 | 0.010 | NA | NA | NA | NA |
| glutamate | C00025 | 0.228 | 0.998 | 0.014 | 0.036 | NA | NA | NA | NA |
| leucine_isoleucine | C00123;  C00407 | 0.228 | 0.998 | 0.011 | 0.010 | NA | NA | NA | NA |
| sn-glycerol-3-phosphate | C00093 | 0.239 | 0.998 | NA | NA | NA | NA | NA | NA |
| 2-oxo-4-methylthiobutanoate | C01180 | 0.271 | 0.998 | 0.029 | NA | NA | NA | NA | NA |
| uracil | C00106 | 0.271 | 0.998 | NA | 0.029 | NA | NA | NA | NA |
| ophthalmate | C21016 | 0.278 | 0.998 | NA | 0.029 | NA | NA | NA | NA |
| arginine | C00062 | 0.309 | 0.998 | 0.001 | 0.003 | NA | NA | NA | NA |
| deoxyuridine | C00526 | 0.321 | 0.998 | 0.008 | NA | NA | NA | NA | NA |
| ornithine | C00077 | 0.321 | 0.998 | 0.001 | 0.004 | NA | NA | NA | NA |

**Supplemental Table 1 continued.** False discovery rate values for the relationship between follicular fluid metabolites and serum estradiol at GnRH2, pre-ovulatory follicle diameter at GnRH2, or final model covariates.

^1^ KEGG = Kyoto Encyclopedia of Genes and Genomes

^2^ E2 = estradiol

^3^ GnRH2 = gonadotropin releasing hormone administration to induce the pre-ovulatory gonadotropin surge

^4^ FF = follicular fluid

^5^ P4 = progesterone

^6^ FA = follicle aspiration

^7^ BCS = Body condition score

^8^ Pubmed chem ID

^1^ KEGG = Kyoto Encyclopedia of Genes and Genomes

^2^ E2 = Estradiol

^3^ GnRH2 = Gonadotropin releasing hormone administration to induce the pre-ovulatory gonadotropin surge

^4^ FF = Follicular fluid

^5^ P4 = Progesterone

^6^ FA = Follicle aspiration

^7^ BCS = Body condition score

| **Metabolite** | **KEGG ID^1^** | **False Discovery Rate** | | | | | | | |
| --- | --- | --- | --- | --- | --- | --- | --- | --- | --- |
|  |  | **Serum E2^2^ at GnRH2^3^, pg/ml** | **Follicle diameter at GnRH2, mm** | **FF^4^ P4^5^ at FA^6^, ng/ml** | **Weight, kg** | **Hours GnRH2 to FA** | **BCS^7^** | **FF E2 at FA, ng/ml** | **Days postpartum** |
| cystathionine | C02291 | 0.324 | 0.998 | 0.011 | NA | NA | NA | NA | NA |
| 6-phospho-d-gluconate | C00345 | 0.335 | 0.998 | NA | NA | NA | NA | NA | NA |
| citrulline | C00327 | 0.350 | 0.998 | 0.001 | 0.004 | NA | NA | NA | NA |
| cysteine | C00097 | 0.380 | 0.998 | NA | NA | NA | NA | NA | NA |
| guanidoacetic acid | C00581 | 0.430 | 0.998 | 0.006 | NA | NA | NA | NA | NA |
| taurine | C00245 | 0.432 | 0.998 | NA | NA | NA | NA | NA | NA |
| uric acid | C00366 | 0.432 | 0.998 | 0.011 | 0.008 | NA | NA | NA | NA |
| trehalose_sucrose | C01083;  C00089 | 0.472 | 0.998 | NA | NA | NA | NA | NA | NA |
| methionine sulfoxide | C02989 | 0.505 | 0.998 | 0.003 | 0.007 | NA | NA | NA | NA |
| hydroxyphenylacetate | C05852 | 0.516 | 0.998 | 0.046 | NA | NA | 0.037 | NA | NA |
| cholesterol sulfate | C18043 | 0.523 | 0.998 | NA | 0.008 | 0.0163 | NA | NA | NA |
| orotate | C00295 | 0.545 | 0.998 | NA | 0.037 | NA | NA | NA | NA |
| octulose bisphosphate | --- | 0.588 | 0.998 | NA | NA | NA | NA | NA | NA |
| 2-3-dihydroxybenzoate | C00230 | 0.598 | 0.998 | NA | NA | NA | 0.042 | NA | NA |
| n-acetylglucosamine-1-6-phosphate | C00357 | 0.663 | 0.998 | NA | NA | NA | NA | NA | NA |
| cystine | C00491 | 0.706 | 0.998 | 0.0138 | 0.031 | NA | NA | NA | NA |
| s-methyl-5-thioadenosine | C00170 | 0.706 | 0.998 | NA | NA | NA | 0.042 | NA | 0.007 |
| gmp | C00144 | 0.716 | 0.998 | NA | NA | NA | NA | NA | NA |
| biotin | C00120 | 0.742 | 0.998 | 0.019 | NA | NA | NA | NA | NA |
| lactate | C00186 | 0.742 | 0.998 | NA | NA | NA | NA | NA | NA |
| myo-inositol | C00137 | 0.742 | 0.998 | NA | NA | NA | NA | NA | NA |
| phosphorylethanolamine | C00346 | 0.742 | 0.998 | NA | NA | NA | NA | NA | NA |
| quinolinate | C03722 | 0.742 | 0.998 | NA | NA | NA | NA | NA | NA |
| aspartate | C00049 | 0.755 | 0.998 | NA | NA | NA | NA | NA | NA |
| xanthurenic acid | C02470 | 0.794 | 0.998 | NA | NA | NA | NA | NA | NA |
| phenyllactic acid | C01479 | 0.823 | 0.998 | NA | NA | NA | NA | NA | NA |
| jasmonate | C08491 | 0.968 | 0.998 | NA | NA | NA | NA | NA | NA |
| citraconate | C02226 | 0.992 | 0.998 | NA | NA | 0.016 | NA | NA | NA |

^1^ GnRH2 = Gonadotropin releasing hormone administration to induce the pre-ovulatory gonadotropin surge

^2^ Number of differentially abundant metabolites in pathway/total number of metabolites in pathway

^3^ FDR = false discovery rate

^4^ Displayed as Name (KEGG identifier number); KEGG = Kyoto Encyclopedia of Genes and Genomes

**Supplemental Table 2.** KEGG pathways significantly enriched with metabolites that were significantly associated with serum estradiol concentration at GnRH2^1^, pg/ml.

| **Pathway** | **Pathway Name** | **Match Status^2^** | **FDR^3^** | **Differentially Abundant Metabolites in Pathway^4^** |
| --- | --- | --- | --- | --- |
| bta00970 | Aminoacyl-tRNA biosynthesis | 5 out of 48 | 0.0183 | L-Asparagine (C00152), L-Histidine (C00135), Glycine (C00037), L-Lysine (C00047), L-Proline (C00148) |
| bta00020 | Citrate cycle (TCA cycle) | 3 out of 20 | 0.0705 | α-ketoglutarate (C00026), S-Malate (C00149), Pyruvate (C00022) |

^1^ Number of differentially abundant metabolites in pathway/total number of metabolites in pathway

^2^ FDR = false discovery rate

^3^ Displayed as Name (KEGG identifier number); KEGG = Kyoto Encyclopedia of Genes and Genomes

**Supplemental Table 3.** KEGG pathways significantly enriched with metabolites that were significantly associated with follicular fluid progesterone concentration at follicle aspiration, ng/ml.

| **Pathway** | **Pathway Name** | **Match Status ¹** | **FDR ²** | **Differentially Abundant Metabolites in Pathway ³** |
| --- | --- | --- | --- | --- |
| bta00970 | Aminoacyl-tRNA biosynthesis | 12 of 48 | 4.23E-06 | L-Asparagine (C00152), L-Histidine (C00135), L-Phenylalanine (C00079), L-Arginine (C00062), L-Glutamine (C00064), Glycine (C00135), L-Methionine (C00073), Glycine (C00047), Tryptophan (C00078), L-Tyrosine (C00082), Glycine (C00148), L-Glutamate (C00025) |
| bta00220 | Arginine biosynthesis | 7 of 14 | 7.72E-06 | L-Glutamate (C00025), L-Arginine (C00062), N-Acetylornithine (C00437), L-Citrulline (C00327), L-Ornithine (C00077), L-Glutamine (C00064), α-ketoglutarate (C00026) |
| bta00330 | Arginine and proline metabolism | 8 of 38 | 0.001 | L-Arginine (C00062), Guanidinoacetate (C00581), Creatine (C00300), Hydroxyproline (C01157), Glycine (C00148), L-Glutamate (C00025), L-Ornithine (C00077), Pyruvate (C00022) |
| bta00250 | Alanine, aspartate and glutamate metabolism | 6 of 28 | 0.008 | L-Asparagine (C00152), L-Glutamate (C00025), L-Glutamine (C00064), Pyruvate (C00149), N-Carbamoyl-L-aspartate (C00438), α-ketoglutarate (C00026) |
| bta00471 | D-Glutamine and D-glutamate metabolism | 3 of 5 | 0.008 | L-Glutamate (C00025), L-Glutamine (C00064), α-ketoglutarate (C00026) |
| bta00630 | Glyoxylate and dicarboxylate metabolism | 6 of 32 | 0.012 | cis-Aconitate (C00417), S-Malate (C00149), Glycine (C00135), L-Glutamate (C00025), Pyruvate (C00149), L-Glutamine (C00064) |
| bta00270 | Cysteine and methionine metabolism | 6 of 33 | 0.012 | 4-Methylthio-2-oxobutanoic acid (C01180), L-Cystathionine (C02291), L-Homocysteine (C00155), L-Methionine (C00073), L-Cystine (C00491), Pyruvate (C00149) |
| bta00240 | Pyrimidine metabolism | 6 of 38 | 0.023 | L-Glutamine (C00064), Uridine (C00299), Cytidine (C00475), Deoxycitidine (C00881), DeoxyUridine (C00299), N-Carbamoyl-L-aspartate (C00438) |
| bta00020 | Citrate cycle (TCA cycle) | 4 of 20 | 0.049 | α-ketoglutarate (C00026), S-Malate (C00149), cis-Aconitate (C00417), Pyruvate (C00149) |
| bta00260 | Glycine, serine and threonine metabolism | 5 of 34 | 0.058 | Guanidinoacetate (C00581), L-Cystathionine (C02291), Glycine (C00135), Creatine (C00300), Pyruvate (C00149) |
| bta00400 | Phenylalanine, tyrosine, and tryptophan biosynthesis | 2 of 4 | 0.058 | L-Phenylalanine (C00079), L-Tyrosine (C00082) |
| bta00360 | Phenylalanine metabolism | 3 of 12 | 0.058 | L-Phenylalanine (C00079), 2-Hydroxyphenylacetate (C05852), L-Tyrosine (C00082) |

^1^ Number of differentially abundant metabolites in pathway/total number of metabolites in pathway

^2^ FDR = false discovery rate

^3^ Displayed as Name (KEGG identifier number); KEGG = Kyoto Encyclopedia of Genes and Genomes

**Supplemental Table 4.** KEGG pathways significantly enriched with metabolites that were significantly associated with cow weight at follicle aspiration, kg.

| **Pathway** | **Pathway Name** | **Match Status ¹** | **FDR ²** | **Differentially Abundant Metabolites in Pathway ³** |
| --- | --- | --- | --- | --- |
| bta00220 | Arginine biosynthesis | 7 of 14 | 1.04E-05 | L-Glutamate (C00025); L-Arginine (C00062); N-Acetylornithine (C00437), L-Citrulline (C00327), L-Ornithine (C00077), L-Glutamine (C00064); α-ketoglutarate (C00026) |
| bta00970 | Aminoacyl-tRNA biosynthesis | 11 of 48 | 1.20E-05 | L-Asparagine (C00152), L-Phenylalanine (C00079); L-Arginine (C00062); L-Glutamine (C00064), Glycine (C00037), L-Methionine (C00073), L-Lycine (C00047), L-Tryptophan (C00078), L-Tyrosine (C00082); L-Proline (C00148); L-Glutamate (C00025) |
| bta00330 | Arginine and proline metabolism | 7 of 38 | 0.007 | L-Arginine (C00062), Creatine (C00300), Hydroxyproline (C00148); L-Proline (C00148), L-Glutamate (C00025), L-Ornithine (C00077), Pyruvate (C00022) |
| bta00471 | D-Glutamine and D-Glutamate metabolism | 3 of 5 | 0.008 | L-Glutamate (C00025); L-Glutamine (C00064); α-ketoglutarate (C00026) |
| bta00020 | Citrate cycle (TCA cycle) | 5 of 20 | 0.008 | α-ketoglutarate (C00026), S-Malate (C00149); cis-Aconitate (C00417), Pyruvate (C00022), Phosphoenolpyruvate (C00074) |
| bta00630 | Glyoxylate and dicarboxylate metabolism | 6 of 32 | 0.009 | cis-Aconitate (C00417), S-Malate (C00149), Glycine (C00037), L-Glutamate (C00025), Pyruvate (C00022), L-Glutamine (C00064) |
| bta00240 | Pyrimidine metabolism | 6 of 38 | 0.019 | L-Glutamine (C00064), Uridine (C00299), Cytidine (C00475), Deoxycytidine (C00475), Orotate (C00295); Uracil (C00106) |
| bta00250 | Alanine, aspartate and glutamate metabolism | 5 of 28 | 0.024 | Asparagine (C00152), L-Glutamate (C00025), L-Glutamine (C00064), Pyruvate (C00022), α-ketoglutarate (C00026) |
| bta00270 | Cysteine and methionine metabolism | 5 of 33 | 0.046 | L-Homocysteine (C00155); L-Methionine (C00073), L-Cystine (C00491), Pyruvate (C00022), Ophthalmate |
| bta00400 | Phenylalanine, tyrosine, and tryptophan biosynthesis | 2 of 4 | 0.058 | L-Phenylalanine (C00079); L-Tyrosine (C00082) |
